# Advanced Electrode Materials Dedicated for Electroanalysis

**DOI:** 10.3390/ma17153762

**Published:** 2024-07-30

**Authors:** Mariola Brycht, Barbara Burnat, Sławomira Skrzypek

**Affiliations:** University of Lodz, Faculty of Chemistry, Department of Inorganic and Analytical Chemistry, Tamka 12, 91-403 Lodz, Poland; slawomira.skrzypek@chemia.uni.lodz.pl

## 1. Introduction

The development of advanced electrode materials has significantly enhanced the capabilities of electrochemical devices, enabling their application in diverse fields such as environmental monitoring, medical diagnostics, food safety, and industrial processes. Ideal electrode materials are expected to exhibit high electrical conductivity and rapid electron transfer across a broad range of redox systems and maintain structural and electrochemical stability over a wide potential range. Additionally, simplicity and low production costs are highly desirable attributes. Meeting these criteria ensures that the electrode material can serve as an effective modification platform for subsequent electroanalytical applications. Numerous types of electrode materials have been developed and successfully applied in electroanalysis, finding applications in environmental, healthcare, and pharmaceutical analyses as electrochemical detectors, micro-/nano-electrochemical devices, and chemical and biochemical sensors. Given their broad applicability, there is a constant demand for innovative advanced electrode materials that offer high selectivity, sensitivity, operational simplicity, low production cost, and miniaturization potential.

This Special Issue, entitled “Advanced Electrode Materials Dedicated for Electroanalysis”, brings together high-quality feature papers that provide insights into and highlight the latest progress and innovative developments in electrode materials. The topics covered include the fabrication and processing of various advanced electrode materials as well as their characterization and potential electrochemical applications.

## 2. Advanced Electrode Materials Discussed in Articles Published in This Special Issue

The collection of eleven articles featured in this Special Issue reflects the significant progress and innovation in the development and application of electrode materials for electrochemical analysis. These papers can be divided into seven categories according to their contents: surface-modified electrodes, bulk-modified electrodes, microelectrodes, biosensors, molecular recognition systems, electrodes for electrocatalytic systems, and solid-contact ion selective electrodes.

### 2.1. Surface-Modified Electrodes

Surface modifications of electrodes can significantly enhance their electrochemical performance, making them highly effective for various analytical applications. These modifications typically involve the application of different materials to the electrode surface to improve sensitivity, selectivity, and overall performance. Common techniques for surface modification include drop-casting, electrodeposition, and layer-by-layer assembly. Among these, drop-casting is the most commonly used technique.

The article by Bargiel et al. [1] presents the development of an electrochemical sensor based on a glassy carbon electrode (GCE) modified with a hybrid material composed of carbon black nanoparticles and Nafion. This drop-casting modification, combined with a preconcentration step, significantly increases the active surface area and improves the limit of detection (LOD) and sensitivity for the beta blocker drug propranolol. The high accuracy and potential for routine laboratory use were validated with real sample analyses, including pharmaceutical tablets and human urine. Praised for its simplicity, low cost, and excellent analytical performance, the method outperforms many existing techniques, such as spectrophotometry and high-performance liquid chromatography (HPLC), in terms of LOD and sensitivity. These findings suggest that the developed voltammetric method is a valuable tool for propranolol determination in both pharmaceutical and biological samples.

The work by Wasąg and Grabarczyk [2] presents the development of two electrochemical sensors for the anodic stripping voltammetric determination of ultra trace concentrations of Cd(II) in environmental water samples. These sensors include a GCE and carbon nanotubes modified screen-printed electrode (CN/SPE), both with an electrochemically deposited copper film (CuF). The modified CuF/CN/SPE, along with an accumulation step, demonstrated a slightly lower LOD and better performance in terms of sensitivity and field applicability. The method was validated with certified reference materials and real water samples, demonstrating the electrodes’ suitability for environmental monitoring. Both sensors showed satisfactory reproducibility and selectivity. These results suggest that both developed electrodes are effective tools for the sensitive and accurate determination of Cd(II) in various environmental contexts and offer a non-toxic alternative to traditional mercury electrodes.

The study by Festinger et al. [3] on graphene oxides as electrode surface modifiers reveals the complexity and variability of these materials. In this work, noble metal (gold and platinum) disk electrodes were modified by drop-casting suspensions of two types of graphene oxides (GO I and GO II) at different concentrations. The resulting surface-modified electrodes were characterized for their spectral, structural, and electrochemical properties. Despite having similar topographies, elemental analysis and electrochemical studies revealed significant differences in the oxygen content and performance between the two types of graphene oxide tested. Surprisingly, the authors found that GO I, acquired as graphene oxide, is more reduced than GO II, which was purchased as reduced graphene oxide. This study underscores the need to standardize graphene oxide-based materials to ensure repeatability and reliability in electrochemical applications.

### 2.2. Bulk-Modified Electrodes

Bulk modifications involve incorporating functional materials into the bulk of the electrode to improve its overall properties. These modifications can significantly enhance the electrochemical performance by increasing the effective surface area and improving electron transfer capabilities. Compared to surface modifications, bulk modifications often provide more robust and stable enhancements, as the active materials are distributed throughout the electrode rather than just on the surface. This can lead to improved durability and longer lifespan of the electrode.

Monteiro et al. [4] developed a ratiometric electrochemical sensor based on a carbon paste electrode (CPE) bulk-modified with a quinazoline-engineered Prussian blue analogue (qnz-PBA) for the determination of the herbicide butralin. This sensor utilizes the stable signal of qnz-PBA as an internal reference to minimize deviations across multiple assays, thus enhancing the precision and accuracy of the measurements. The performance of the CPE modified with qnz-PBA was successfully validated using lettuce and potato samples, confirming its accuracy and applicability in real-world scenarios. This ratiometric sensor offers simplicity, cost-effectiveness, and excellent analytical performance. The authors stated that the developed ratiometric sensor is an effective tool for the sensitive and precise determination of butralin in both agricultural and environmental samples and is a reliable and accurate alternative to chromatographic methods used for butralin determination.

The work by Brycht et al. [5] presents the development of a carbon ceramic electrode (CCE) bulk-modified with bismuth(III) oxide nanoparticles (Bi_2_O_3_NPs) for the determination of 4-chloro-3-methylphenol (PCMC), a priority environmental pollutant. The CCE modified with Bi_2_O_3_NPs (Bi-CCE), characterized using microscopic and electrochemical techniques, demonstrated a more compact and less porous surface compared to the unmodified CCE and a higher effective surface area, indicating an increased number of electroactive sites. Additionally, the incorporation of Bi_2_O_3_NPs significantly enhances the electrochemical properties of the CCE, providing an extended linear detection range, improved sensitivity and a lower LOD compared to the unmodified CCE. The presence of Bi_2_O_3_NPs improves electron transfer and reduces background current. The sensor’s performance was validated using river water samples, demonstrating excellent recovery rates and selectivity. The Bi-CCE exhibited high reproducibility and stability over three months. These results suggest that the Bi-CCE is an effective tool for the sensitive and accurate determination of PCMC and other phenolic compounds, with potential applications in environmental monitoring and pollutant detection.

### 2.3. Microelectrodes

Microelectrodes offer unique advantages due to their small size, including enhanced mass transfer rates and reduced ohmic losses. These properties make them highly suitable for sensitive and precise analytical applications. Their small dimensions lead to a higher current density and faster response times compared to macroelectrodes, making them ideal for real-time monitoring and detection in confined environments.

Araújo et al. [6] present the development of a homemade carbon fiber microelectrode (CF-µE) for quantifying caffeine in soft beverages. The fabricated microelectrode, characterized for its electrochemical properties, demonstrated significantly enhanced sensitivity and a lower LOD for caffeine compared to other analytical methods. The CF-µE exhibited a high mass transfer rate and a sigmoidal voltammetric profile, confirming its microelectrode characteristics. The sensor’s stability and reproducibility were confirmed over multiple tests. The method was validated with real soft beverage samples, showing satisfactory results consistent with the literature values and HPLC validation. The homemade CF-µE offers a cost-effective, portable, and reliable alternative for caffeine determination in the beverage industry, highlighting its potential for broader application in quality control and environmental monitoring.

### 2.4. Biosensors

Biosensors incorporate biological elements, such as enzymes, to provide high specificity for target analytes. These sensors are particularly useful for detecting biologically relevant compounds in complex matrices. The integration of biological recognition elements with electrochemical transducers allows for the selective and sensitive detection of various analytes, making biosensors highly valuable in medical diagnostics, environmental monitoring, and food safety.

Krzyczmonik et al. [7] developed an electrochemical enzyme-based biosensor for the determination of polyphenols. The biosensor, constructed on a GCE, utilized a composite material consisting of poly(3,4-ethylenedioxy-thiophene), poly(4-lithium styrenesulfonic acid), chitosan, gold nanoparticles (AuNPs), and glutaraldehyde, which was further modified by immobilizing laccase using glutaraldehyde as a cross-linker. The composite material demonstrated boosted electrical conductivity, enhanced stability of the chitosan layer while maintaining high biocompatibility, and improved surface morphology, confirmed through wide range of complementary analytical techniques. The addition of AuNPs increased the effective surface area and facilitated the oxidation of polyphenols. The biosensor exhibited high catalytic activity and excellent performance towards the oxidation and detection of polyphenols such as catechol, gallic acid, and caffeic acid. The practical applicability of the developed biosensor was validated using white wine samples. This innovative biosensor offers a cost-effective, highly sensitive, and stable alternative for polyphenol determination in various environmental and biological samples.

### 2.5. Molecular Recognition Systems

The development of molecular recognition systems has become a primary objective in modern electrochemistry. These systems, which play a crucial role in various analytical applications, are created by depositing molecules with specific properties onto the surfaces of electrode materials. Typically, semiconductors (such as silicon) or dielectrics (such as glass or ceramics) are used as the base materials, which acquire valuable properties by forming conductive or semiconductive structures on their surfaces. Most commonly, a chemically defined layer of inorganic oxides or carbon materials with distinct electrical properties is applied to the surface of the base material.

The work by Cirocka et al. [8] presents the search for optimal electrode materials to serve as platforms for future sensors. A wide group of electrode materials was tested, including fluorine-doped tin oxide (FTO), silicon modified with carbon nanowalls, and silicon and glass modified with nanocrystalline boron-doped diamond layers with varying B/C ratios. The electrochemical properties and wettability of these electrode materials were evaluated and compared to commercially available carbon-based electrodes, such as boron-doped diamond electrode and GCE. Among the tested electrode materials, FTO was identified as the optimal electrode material due to its excellent electrochemical properties, high chemical stability, and valuable optoelectronic characteristics, making it a prime candidate for further research and development in various analytical and industrial applications.

The study by Domaros et al. [9] on the modification of transparent conductive oxide electrodes with alkoxysilanes demonstrates the potential for creating selective molecular recognition systems. In this work, FTO electrodes were silanized using 3-aminopropyltrimethoxysilane, trimethoxy(propyl)silane, and trimethoxy(octyl)silane under various reaction conditions (time and temperature). The modification process included single and double alkoxysilane modifications, as well as two-step mixed alkoxysilane modifications. The obtained electrodes were characterized in terms of electrochemical properties and wettability. The research highlights how different alkoxysilane structures and modification conditions can control surface properties and charge transfer processes, paving the way for tailored analytical tools with enhanced selectivity and sensitivity.

### 2.6. Electrodes for Electrocatalytic Systems

Electrodes play a crucial role in electrocatalytic systems, serving as the interface where electrochemical reactions occur. Their importance lies in their ability to facilitate electron transfer, which is essential for efficiently catalyzing reactions. The development of novel electrode materials aims to optimize these systems for better performance, efficiency, and durability in a wide range of applications, including fuel cells, electrolyzers, batteries, and electrochemical sensors. A specific emphasis is placed on the electrocatalytic oxidation of small organic compounds, such as methanol, ethanol, isopropanol, formaldehyde, and formic acid, on various modified electrodes. Many electrocatalytic systems employ noble metals such as platinum and palladium as key components, due to their unique catalytic properties.

Leniart et al. [10] report on the development of an advanced electrochemical platform based on a GCE modified with multi-walled carbon nanotubes (MWCNTs) and palladium nanoparticles (PdNPs). The MWCNTs were applied to the GCE surface using the drop-casting method, while PdNPs were produced electrochemically via a potentiostatic method using various programmed charges from an ammonium tetrachloropalladate(II) solution. This charge-controlled electrodeposition method enabled precise control over the amount and size of the deposited PdNPs. Detailed characterization and electrochemical assessment of GCE/MWCNTs/PdNPs revealed that the size and dispersion of PdNPs significantly influence the catalytic activity towards formaldehyde oxidation. Additionally, the long-term stability of the modified electrodes highlights their potential for practical applications.

### 2.7. Solid-Contact Ion Selective Electrodes

Solid-contact ion-selective electrodes (SC-ISEs) represent a significant advancement in the field of ion detection, offering improved performance, stability, and versatility across multiple applications. SC-ISEs are a type of ion-selective electrode that utilize a solid-state material as the transducer element between the ion-selective membrane and the electron-conducting substrate. Unlike traditional ISEs that rely on liquid-filled internal solutions, SC-ISEs incorporate solid-contact layers, which can be conducting polymers, carbon-based materials, etc. However, the search for the ideal transducer material is still ongoing. The perfect transducer material should exhibit a reversible transition from ionic to electronic conduction, high exchange current density, stable chemical composition, and possibly high hydrophobicity to minimize the formation of water at the transducer-membrane interface. Recently, the use of metal–organic frameworks (MOFs), a sub-class of highly ordered and porous materials with two- or three-dimensional structures, seems to be promising material as an ion-to-electron transducer.

The article of Kościelniak et al. [11] describes preliminary studies on the development of a solid-contact ion-selective electrode for detecting potassium in environmental water. The authors implemented two versions of a stable cadmium acylhydrazone-based MOF (JUK-13 and JUK-13_H_2_O), differing in guest molecules, as the ion-to-electron transducers. Both MOFs significantly improved the potentiometric response and stability of the electrode. The K-JUK-13_H_2_O-ISE demonstrated a good Nernstian slope and excellent long-term potential stability, making it a reliable tool for environmental water analysis. Its successful application in determining potassium in certified reference materials highlights its precision and accuracy.

## 3. Conclusions

The collection of articles featured in this Special Issue reflects the significant progress and innovation in the development and application of electrode materials for electrochemical analysis. The studies presented here offer deep insights into surface-modified electrodes, bulk-modified electrodes, microelectrodes, biosensors, molecular recognition systems, electrodes for electrocatalytic systems, and solid-contact ion selective electrodes, highlighting the interdisciplinary nature of this field. Collectively, the articles published within this Special Issue emphasize the importance of electrode material selection, surface modification, and comprehensive characterization in advancing electroanalysis. We hope that the findings and discussions presented here will inspire further research and innovation in developing advanced electrode materials for electrochemical applications. As the Guest Editors, we would like to extend our gratitude to all the authors, reviewers, and the editorial team for their contributions to this Special Issue. We believe that the knowledge shared within these pages will significantly impact the future of electrochemical analysis and its applications.
